# Talking with pediatric patients with overweight or obesity and their parents: self-rated self-efficacy and perceived barriers of Dutch healthcare professionals from seven disciplines

**DOI:** 10.1186/s12913-022-08520-2

**Published:** 2022-10-06

**Authors:** B. van der Voorn, R. Camfferman, J. C. Seidell, J. Halberstadt

**Affiliations:** Faculty of Science, Department of Health Sciences, Vrije Universiteit Amsterdam, Section Youth and Lifestyle, De Boelelaan 1085, 1081 HV Amsterdam, The Netherlands

**Keywords:** Communication barriers, Health communication, Pediatric obesity, Netherlands, Overweight

## Abstract

**Background:**

Many healthcare professionals (HCPs) feel uncomfortable and incompetent talking about weight with children with overweight and obesity and their parents. To optimally target interventions that can improve obesity care for children, we assessed the self-efficacy (SE) and perceived barriers (PBs) of Dutch HCPs with regard to talking about weight and lifestyle when treating children with overweight or obesity. We also analyzed interdisciplinary differences.

**Methods:**

A newly developed, practice- and literature-based questionnaire was completed by 578 HCPs from seven disciplines. ANOVA and chi-square tests were used to analyze interdisciplinary differences on SE, PBs, and the effort to discuss weight and lifestyle despite barriers. Regression analyses were used to check whether age, sex or work experience influenced interdisciplinary differences.

**Results:**

On average, the reported score on SE was 7.2 (SD 1.2; scale 1–10) and the mean number of PBs was 4.0 (SD 2.3). The majority of HCPs (94.6%) reported perceiving one or more barriers (range 0–12 out of 17). HCPs who in most cases perceived too many barriers to discuss weight and lifestyle of the child (9.6%, *n* = 55) reported a lower SE (mean 6.3) than professionals who were likely to discuss these topics (mean SE 7.3, *p* < 0.01), despite having a similar number of PBs (mean 4.5 vs 4.0, *p* > 0.05). In total, 14.2% (*n* = 82) of HCPs either felt incapable (SE ≤ 5) or reported that in most cases they did not address weight and lifestyle due to PBs.

**Conclusions:**

Although on average Dutch HCPs rated their self-efficacy as fairly good, for a subgroup major improvements are necessary to lower perceived barriers and improve self-efficacy, in order to improve the quality of care for Dutch children with obesity.

**Supplementary Information:**

The online version contains supplementary material available at 10.1186/s12913-022-08520-2.

## Background

In 2020, as many as 14.7% of Dutch children (ages 4–18) had overweight, of which 2.5% had obesity [1]. To discuss weight and lifestyle effectively with these children and their primary caregivers (in most cases parents), healthcare professionals (HCPs) are required to have specific knowledge, skills and attitudes [2–5]. However, many HCPs report feeling uncomfortable and incompetent talking about weight and lifestyle, and often avoid weight-related topics or refer to them only indirectly [5–7], which might reflect low self-efficacy (SE) [8] or limited communication skills. In addition to these internal barriers, external barriers (i.e. organizational and societal ones) also interfere with optimal obesity care for children [6, 7, 9]. Therefore, studies advocate investing in obesity education programs, along with organization of a suitable financial and infrastructural framework for adequate long-term obesity care [9–11].

Perceived SE is influenced by multiple internal and external factors, including how individuals perceive their knowledge, previously acquired skills, emotional wellbeing, task complexity, professional role and available time [8, 12, 13]. In this study, SE refers to a person’s perceived capability to successfully address weight and lifestyle when treating children with overweight or obesity and their parents. A person assesses their SE by analyzing the internal and external factors that are involved in their task-specific capability, i.e. a combination of perceived barriers (PBs) [8, 12]. Although behavior is influenced by more factors than SE alone, such as motivation, SE can predict a person’s intentions and moderates behavioral choices and commitment [8, 12]. In other words, SE represents the drive to initiate and adapt behavior, aiming to match one’s performance with the circumstances. Accordingly, an increased SE, which can result from training [14–16], could help HCPs overcome the barriers they perceive. However, how reported self-efficacy is associated with perceived barriers and the effort to start the conversation has not been studied, and insights into interdisciplinary differences are lacking.

To optimally target interventions that can improve obesity care for children, we assessed the SE and PBs of Dutch HCPs that treat children with overweight or obesity, with respect to talking about weight and lifestyle. We also analyzed interdisciplinary differences.

## Methods

### Aim, design and setting of the study

The aim of this study was to assess how HCPs working within different levels of pediatric obesity care perceive their self-efficacy and the barriers they face when talking about weight and lifestyle with children with overweight or obesity and their parents. We assessed whether these scores affected the effort they put into starting the conversation despite barriers. This observational, self-report study was conducted nationwide in the Netherlands in 2018. HCPs working with children and adolescents with overweight or obesity were recruited through their professional associations, at conferences, via personal contacts, and through public health services.

### Participants and procedure

Seven disciplines of HCPs were included: general practitioners (GPs), youth healthcare physicians (YHCPs), youth healthcare nurses (YHCNs), pediatricians, mental health professionals (including child psychologists, pedagogic HCPs and nurse practitioners specialized in mental health care), dieticians and physiotherapists. Together these seven disciplines represent all HCPs that work within the Dutch National model of integrated care for pediatric obesity. In total, 633 professionals from all levels of obesity care returned the completed questionnaire. For this study we included participants that worked in the seven professions listed above (excluding *n* = 19 profession unknown and *n* = 36 community workers), leaving a total of 578 HCPs.

## Measurements

### Self-efficacy

SE was self-reported with a score between 1 and 10 on the question: “How confident do you feel in discussing weight and lifestyle with children and their parents?” [8]. A score of 1 represented low self-efficacy; 10 represented high self-efficacy. In the Netherlands, everybody is familiar with a grading system from 1 to 10 where grade 6 and higher is a pass and grade 5 and lower is a fail. Accordingly, in this study a grade ≤ 5 means that someone feels incapable of performing the task.

### Perceived barriers

A new questionnaire was developed based on the most frequently reported barriers in literature, to ask what barriers HCPs perceived when discussing weight and lifestyle with children with overweight or obesity and their parents. Supplementary Table 1 summarizes the 16 barriers included. HCPs were asked which of the barriers were applicable in their work. The barriers were scored dichotomously: 0 = not applicable, 1 = applicable. HCPs were likewise given the opportunity to provide an additional barrier that was not included in the questionnaire. A maximum of 17 barriers could be reported, 16 of them presented as a single-choice question and one open-ended.

### Avoidance of the topic

HCPs were asked how often they avoid discussing weight and lifestyle due to PBs when they interact with a child with overweight or obesity and their parents. The following multiple-choice answers were offered: 1 = Never, I always discuss weight; 2 = In less than a quarter of the cases I do not discuss weight; 3 = In about half of the cases I do not discuss weight; 4 = In three quarters of the cases I do not discuss weight; and 5 = I never discuss weight. Answers were subsequently dichotomized: 0 = Almost always discusses weight and lifestyle (options 1 and 2) and 1 = Does not frequently discuss weight and lifestyle (options 3, 4, and 5).

### Statistical analyses

For the statistical analyses, IBM SPSS 27.0 was used. First, descriptive statistics were conducted to summarize the reported score on SE and number of PBs for each group of HCPs separately and for the total group. Second, ANOVA with Bonferroni correction was computed to assess interdisciplinary differences on reported SE and number of PBs. Third, frequencies of each barrier were computed for each professional group and the total group of professionals. Fourth, Fisher’s exact tests were calculated to assess the relationship between discussing weight (almost always vs not frequently) and each PB (not applicable vs applicable), and Chi Square tests were used to assess interdisciplinary differences on avoidance of the topic. Fifth, a t-test was done to assess the difference between the HCPs who do not frequently discuss weight and HCPs who almost always discuss weight on their reported SE. Sixth, the influence of confounding factors was investigated using multivariate regression, with SE or PBs as dependent factor and profession (in dummy variables with pediatricians first and mental health professionals second as comparison group), sex, age, and years in profession as independent factors. Last, the barriers that the HCPs provided in the open-ended question were scored into categories by two coders that aimed to remain as close as possible to the text.

## Results

Table 1 summarizes the characteristics of 578 participants. The number of missing determinants was low: sex was missing for 13 participants (2.2%), age for 5 (0.9%), and years in profession for 5 (0.9%). The male–female ratio differed significantly (*p* < 0.01) between groups, with the largest percentage of males among pediatricians (33.3%, *n* = 8) and the lowest among YHCNs (1.2%, *n* = 3). A significant (*p* < 0.01) age difference was found, with pediatricians being the oldest (mean age [SD] 50.5 [9.2] years) and dieticians the youngest (37.6 [13.2] years). HCPs’ professional experience differed significantly (*p* = 0.01), with physiotherapists having the most experience (median [IQR] 17.0 [6.5–27.5] years) and GPs having the least (5.5 [2.6–18.3] years).

**Table 1:** Descriptives of the sample

		**Total group**	**Pediatricians**	**Dieticians**	**YHCPs**	**Mental health professionals**	**YHCNs**	**Physiotherapists**	**GPs**
**N**		**578**	**31**	**78**	**92**	**56**	**260**	**41**	**25**
**Males**	N (%)	36 (6.2%)	8 (25.8%)	3 (3.8%)	9 (9.8%)	3 (5.4%)	3 (1.1%)	4 (9.8%)	6 (24.0%)
**Age in years**	Mean (SD)	43.2 (12.0)	50.5 (9.2)	37.6 (13.2)	45.2 (11.2)	42.8 (11.9)	43.4 (11.9)	41.9 (12.1)	43.0 (10.8)
**Years in profession**	Median [IQR]	11.0 [5.0–20.0]	16.0 [10.0–25.0]	8.0 [2.0–22.0]	12.5 [6.0–25.0]	12.0 [4.8–20.0]	11.0 [4.5–20.0]	17.0 [6.5–27.5]	5.5 [2.6–18.3]

*GPs* general practitioners, *YHCPs* youth healthcare physicians, *YHCNs* youth healthcare nurses, *SD* standard deviation, *IQR* interquartile range

### Self-efficacy

Scores on SE were known for 554 HCPs (95.8%) and are displayed in Table 2. The mean score of the different groups of professionals ranged from 6.8 to 8.1 (overall mean 7.2 [SD 1.2]). A significant interdisciplinary difference was found (*p* < 0.001): pediatricians reported significantly higher scores on SE compared to all other disciplines, except for dieticians and YHCPs (see Supplementary Table 2A). Adjusting for sex, age, and years in profession did not change this. In addition, 38 HCPs (6.9% of the total group) felt incapable (i.e. rated their SE ≤ 5) of addressing weight and lifestyle with children with overweight or obesity and their parents.

**Table 2:** Perceived self-efficacy and number of different barriers

		**Total group**	**Pediatricians**	**Dieticians**	**YHCPs**	**Mental health professionals**	**YHCNs**	**Physiotherapists**	**GPs**
**Self-efficacy**	Mean (SD)	7.2 (1.2)	8.1 ^*^ (1.2)	7.5 (1.1)	7.4 (1.1)	7.2 (1.7)	7.1 (1.0)	6.9 (1.5)	6.8 (1.6)
	Rated ≤ 5	6.9%	3.2%	2.8%	5.5%	13.0%	6.5%	12.2%	12.0%
**Number of barriers** Mean (SD)		4.0 (2.3)	2.3 ^*^ (2.0)	3.8 (2.1)	4.7 (2.7)	2.9 ^*^ (2.0)	4.2 (2.1)	4.4 (2.7)	4.4 (2.5)
**Do not discuss weight in ≥ 50% of cases**		9.6%	12.9% ^*^	5.3%^*^	5.4% ^*^	18.0%	2.3% ^*^	30.0%	60%

Self-efficacy was rated on a scale of 0–10. Groups of HCPs were ranked in order of mean perceived SE rating. A maximum of 17 perceived barriers could be reported. Interdisciplinary differences were analyzed post-hoc with Bonferroni correction and significance (*p* < 0.05) marked by an asterisk

*GPs* general practitioners, *YHCPs* youth healthcare physicians, *YHCNs* youth healthcare nurses, *SD* standard deviation, *IQR* interquartile range

### Perceived barriers

**Fig. 1:**
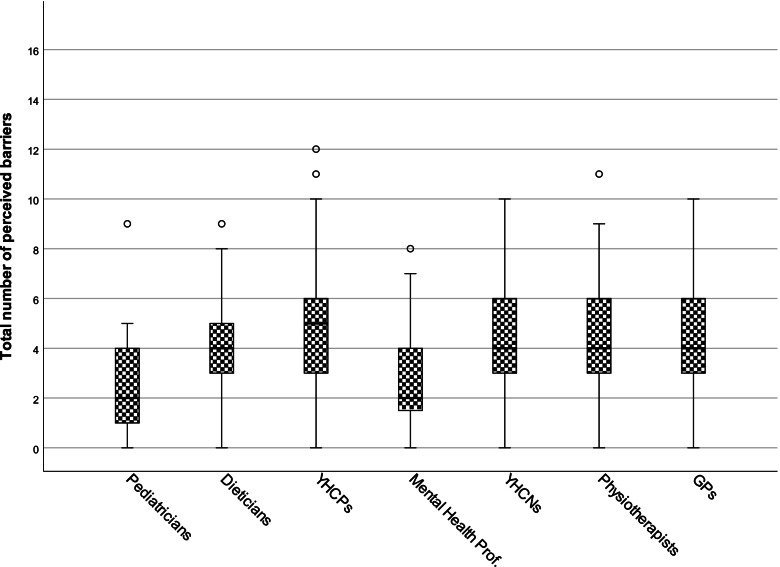
Boxplot of interdisciplinary differences in perceived barriers. HCP groups were ranked in order of perceived self-efficacy rating. A maximum of 17 barriers could be reported

The number of reported PBs was known for all 578 HCPs and ranged between 0 and 12, mean 4.0 (see Fig. 1 and Table 2). Pediatricians reported significantly fewer PBs than other disciplines, except for mental health professionals. Mental health professionals perceived significantly fewer barriers than YHCPs, YHCNs and physiotherapists (see Supplementary Table 2B). By adjusting for sex, age and years in profession the differences between pediatricians and dieticians became non-significant. All other interdisciplinary differences remained. The majority of HCPs (94.6%) reported perceiving one or more barriers (range 1–8, see Table 3).

**Table 3:** Perceived barriers by group of professionals

	Total group		**General practitioners**		**Youth Health Care Physicians**		**Youth Health Care Nurses**		**Pediatricians**		**Mental health professionals**		**Dieticians**		**Physiotherapists**	
	N	%	N	%	N	%	N	%	N	%	N	%	N	%	N	%
Insufficient time	215	37%	15	60%	45	49%	95	37%	15	48%	8	16%	30	39%	7	17%
Not stated in my job description	14	2%	1	4%	0	0%	1	0.4%	0	0%	3	6%	3	4%	6	15%
Insufficient care to refer to	137	24%	5	20%	38	41%	61	24%	17	55%	5	10%	7	9%	4	10%
Child or parent with Dutch as second language	253	44%	12	48%	46	50%	126	49%	7	23%	14	28%	33	42%	15	37%
Child or parent with low cognitive abilities	207	36%	11	44%	38	41%	99	38%	8	26%	9	18%	28	36%	14	34%
Not enough training in specific communication strategies	110	19%	7	28%	18	20%	44	17%	1	3%	9	18%	18	23%	13	32%
My own weight	18	3%	1	4%	1	1%	10	4%	0	0%	2	4%	3	4%	1	2%
Not enough knowledge about which words are best to use	86	15%	7	28%	10	11%	34	13%	1	3%	4	8%	16	21%	14	34%
Afraid that discussing the weight of the child will harm the child emotionally	178	31%	6	24%	24	26%	78	30%	3	10%	14	28%	34	44%	19	46%
Difficult to make children and parents realize the impact of overweight/obesity on health	101	18%	3	12%	18	20%	51	20%	0	0%	7	14%	17	22%	5	12%
Negative experiences with discussing weight and lifestyle	99	17%	2	8%	33	36%	52	20%	2	7%	6	12%	2	3%	2	5%
Expectation that the child and/or parent will react negatively	217	38%	12	48%	46	50%	101	39%	5	16%	12	24%	21	27%	20	49%
Discussing weight could stand in the way of having a good relationship with the child or parent	110	19%	6	24%	17	19%	43	17%	2	7%	13	26%	14	18%	15	37%
Discussing weight could be perceived as a negative judgment about the whole family	253	43.8%	12	48%	46	50%	126	49%	7	23%	14	28%	33	42%	15	37%
Parents with overweight or obesity	239	41%	9	36%	39	42%	125	48%	3	10%	13	26%	30	39%	20	49%
Insufficient knowledge about the causes of overweight and obesity	31	5%	1	4%	0	0%	19	7%	0	0%	5	10%	3	4%	3	8%

*N that reported experiencing this barrier*

HCPs were given the opportunity to provide one or more additional PBs or other remarks, and 148 (23.5%) used this opportunity. Some barriers had already been stated in the questionnaire. Additional PBs (ranked by frequency) were: reasons for consultation other than obesity (*n* = 23), parents who do not acknowledge the weight problem (*n* = 15), perceiving parental resistance to the topic (*n* = 12), insufficient knowledge or insufficient skills (e.g. concerning motivational interviewing and not enough experience in general) (*n* = 10), perceiving a lack of motivation in the parents (*n* = 10), family with a non-Dutch culture (*n* = 7), difficulty addressing parenting issues (*n* = 6), previous failed attempts by the professional or other professionals (*n* = 4), individual challenges, no “one size fits all” (*n* = 3), afraid that the child and parent have heard it multiple times already from different professionals (*n* = 3), parents who feel that the professional is interfering with their personal life (*n* = 2), and insufficient confidence that discussing the weight will be successful (*n* = 1).

### Avoidance of the topic

In total, 570 HCPs (98.6%) answered the question of whether they made the effort to discuss weight and lifestyle despite PBs. Overall, 55 HCPs (9.6%) reported that in most cases they did not address weight and lifestyle due to PBs. Large interdisciplinary differences were found. The majority of GPs (60%) reported not addressing this topic when applicable, which was significantly more often than pediatricians, dieticians, YHCNs, YHCPs and mental health professionals. Physiotherapists reported significantly more often that they did not address this topic, compared to dieticians, YHCNs and YHCPs, and mental health professionals significantly more often compared to YHCNs (see Supplementary Table 2C).

HCPs who in most cases did not address weight and lifestyle due to PBs rated their SE significantly lower (mean SE 6.3 [SD 1.40], *p* < 0.001) than other HCPs who were likely to address this topic (mean SE 7.3 [SD 1.15]), yet reported an equal number of PBs (mean 4.5 vs 4.0, respectively, *p* > 0.05). In total, 14.2% (*n* = 82) of all HCPs either rated their SE below 5 or reported that in most cases they did not address weight and lifestyle due to PBs.

The professionals who in most cases did not address weight and lifestyle (*n* = 55, 9.6%) were more likely to report the following PBs than professionals who were likely to address this topic: not part of my job description (*p* = 0.001), not enough training in specific communication strategies (*p* = 0.003), not enough knowledge about which words are best to use (*p* < 0.001), expectation that the child and/or parent will react negatively (*p* = 0.01), discussing weight could stand in the way of having a good relationship with the child or parent (*p* = 0.004), and insufficient knowledge about the causes of overweight and obesity (*p* = 0.02). By contrast, professionals who almost always discuss weight and lifestyle reported more often as PB a child or parent with Dutch as second language (*p* = 0.01) and that discussing weight could be perceived as a negative judgment about the whole family (*p* = 0.01).

## Discussion and conclusion

The aim of the current study was to examine self-efficacy and perceived barriers of Dutch HCPs with regard to talking about weight and lifestyle when treating children with overweight or obesity and their parents, and to assess interdisciplinary differences. On average, HCPs rated their self-efficacy as fairly good (mean SE 7.2). Although only one in 15 (6.9%) rates their self-efficacy ≤ 5 (a fail), almost all HCPs (94.6%) perceive at least one barrier and one in 10 (9.6%) avoids talking about weight and lifestyle due to barriers. Moreover, HCPs from all seven disciplines – overall one in seven (14.2%) – reported feeling incapable of addressing weight and lifestyle when treating children with overweight or obesity. Reported underlying barriers included both internal and external factors.

Interdisciplinary differences were found in our study: pediatricians had the highest SE ratings and lowest number of PBs, in contrast with GPs with the lowest SE ratings and highest numbers of PBs. Large intradisciplinary differences were also found, e.g. pediatricians rated their SE on average a 8.1, yet still one in eight (12.9%) pediatricians perceived too many barriers to address weight and lifestyle when treating children with overweight or obesity. Recent Canadian studies report similar large interindividual differences among groups of pediatricians [16, 17]. It is important to know that the role of pediatricians in the Netherlands is different than in other countries. In other countries pediatricians also provide primary health care [18, 19], while in the Netherlands they are medical specialists and provide only secondary or tertiary care and subsequently tend to see more patients that already suffer from weight-related problems. The work of a pediatrician in other countries is more comparable to our GPs and YHCPs, i.e. HCPs that signal a problem the patient might not yet be aware of. These differences in tasks could lead to interdisciplinary differences in self-efficacy and perceived barriers when addressing the topic, hampering direct comparisons between countries and healthcare systems.

Even though on average SE ratings were relatively high, up to one in seven HCPs felt incapable of talking about weight and lifestyle. When looking into the care network of one single child who suffers from weight problems and will be seen by multiple HCPs along the way, there is a rather high chance that a child with overweight or obesity will come across at least one professional that do not feel secure about their capabilities to talk about weight and lifestyle and avoid the topic when discussing the child’s health. As studies from other countries also report such feelings by HCPs (and those in training), this finding does not seem unique and therefore does not apply exclusively to the Dutch healthcare setting [6, 10].

In the current study both external (i.e. task attributes and complexity, and the organization of health care, as in “insufficient care to refer to”) and internal determinants of self-efficacy (i.e. individual knowledge, skills and personality, as in “not enough knowledge about which words are best to use”) were reported to form a barrier to optimal obesity care for children. Interestingly, in our study we found that HCPs who almost always discuss weight and lifestyle reported external barriers more often, whereas HPCs who perceive too many barriers to discuss this topic reported internal barriers more often and rated their self-efficacy significantly lower. Many HCPs perceived more problems in talking about weight and lifestyle in cases when parents had overweight or obesity, and/or when they expected the child and/or parent to react negatively. This relates to stigmatization of people with obesity that is present among both the general public and HCPs [20, 21], and asks for education in communication strategies to address this sensitive topic [22], thereby improving HCPs’ perceived self-efficacy and reducing barriers [2, 14, 15, 23, 24]. The majority of doctors (GPs, YHCPs and pediatricians) reported that insufficient time is a barrier to talking about weight and lifestyle. This is a commonly reported barrier in other studies too, and asks for changes in the health delivery system [2, 14, 15, 23, 24]. Accordingly, next to education an improved and sustainable financial and infrastructural framework is necessary to reduce the obesity stigma and positively impact HCPs’ perceived self-efficacy and barriers, improving obesity care for children [6, 10, 20, 21].

The current study has some limitations. First, the sample sizes per subgroup of professionals were limited. Especially GPs and pediatricians were underrepresented and differed significantly in terms of male–female ratio and age, compared to the other disciplines. Colleagues from the same discipline though possibly from different levels of care (e.g. mental health professionals working in either primary, secondary or tertiary care) were grouped within a single subgroup, so discipline-specific results should be interpreted with caution. Second, there is a high chance of selection bias, some professionals might have been more inclined to fill in the questionnaire than others, and we cannot rule out that age- and sex-specific differences were too large to be accounted for by multivariate regression analyses. Third, professionals may have given socially desirable answers. Fourth, we asked HCPs to report perceived capabilities and have not evaluated correlated behavior or practical skills. Our study aim was to gain insight into interdisciplinary differences in task-specific capability and how that relates to perceived barriers and behavioral intentions as we targeted to improve the quality of care by influencing the process.

However, we acknowledge that the results from this study cannot be directly translated into behavior, i.e. the quality of obesity care in the Netherlands.

The study also has strengths. First, the inclusion of seven disciplines, covering all levels of obesity care within both the medical and the social domain, offers a broad perspective. In addition, it is the first study to investigate self-efficacy and perceived barriers in the Dutch healthcare setting among HCPs when it comes to talking about weight and lifestyle as part of the treatment of children with overweight or obesity.

To conclude, Dutch HCPs that work in obesity care for children rate their self-efficacy on average as fairly good. However, a child with overweight or obesity still has a high chance of coming across an HCP that feels incapable of addressing weight and lifestyle in a conversation (one in seven, 14.2%, for each time facing a new HCP). Accordingly, to empower HPCs who feel incapable, major improvements in the health delivery system are necessary. For internal barriers, such as lack of communication skills, education can help when implemented as mandatory training for all current and future HCPs working in pediatric obesity care. For external barriers, such as insufficient time, changes in infrastructure and financial recourses seem necessary**.** But foremost, the interdependency of these barriers asks for adequate investments on the entire obesity care framework and is not limited to one topic. The Dutch National model integrated care for childhood overweight and obesity [25, 26] was developed recently and is currently being implemented nationwide. Once fully implemented and financed, this integrated care model and accompanying materials will offer tailored education, practical tools, and the financial and infrastructural prerequisites. To optimally target such improvements, the association between SE ratings, knowledge and skills – the quality of care – needs to be reassessed continuously during these transitions in obesity care. Moreover, additional research will be necessary to investigate why some HCPs perceive barriers yet feel capable of addressing the topic, whereas others avoid the topic even when appropriate and despite a similar number of perceived barriers, and how this might change over time.

## Supplementary Information

**Additional file 1:** 

**Additional file 2:** 

## Data Availability

The datasets generated and analyzed during the current study are not publicly available, but can be obtained from the corresponding author upon reasonable request. Due to the presence of indirect identifiers it is impossible for healthcare professionals of certain subgroups to stay completely anonymous.

## Abbreviations

GP: General practitioner
HCP: Healthcare professional
PB: Perceived barrier
SE: Self-efficacy
YHCP: Youth healthcare physician
YHCN: Youth healthcare nurse
