# Supplementary material for: Talking with pediatric patients with overweight or obesity and their parents: self-rated self-efficacy and perceived barriers of Dutch healthcare professionals from seven disciplines
Source: BMC Health Serv Res. 2022 Oct 6;22:1236. doi: 10.1186/s12913-022-08520-2 (PMC9541008; doi:10.1186/s12913-022-08520-2)
Supplement: Supplementary file 1 — Additional file 1: [file 12913_2022_8520_MOESM1_ESM.docx]

| **Perceived Barrier that was included in the questionnaire** | **Based on the following literature** |
| --- | --- |
| Insufficient time | (Holt et al., 2011; Jelalian et al., 2003; Steele et al., 2011; Kubik et al., 2007; Turner et al., 2016; Freedman and Stern, 2004; Brink-Melis et al., 2012) |
| Is not stated in my job description | (Redsell et al., 2011; Moyers et al., 2005; Price et al., 1987) |
| Insufficient care to refer to | (Jelalian et al., 2003; Turner et al., 2016; Brink-Melis et al., 2012) |
| Child or parent with Dutch as a second language. | (Quelly, 2014) |
| Child or parent with low cognitive abilities | (McPherson et al., 2017) |
| Not enough training in specific communication strategies | (Gerards et al., 2012; Jelalian et al., 2003; McPherson et al., 2017; Redsell et al., 2011; Turner et al., 2016; Visser et al., 2008) |
| My own weight | (Steele et al., 2011; Moyers et al., 2005; Price et al., 1987) |
| Not enough knowledge about which words are best to use | (Gerards et al., 2012; Jelalian et al., 2003; McPherson et al., 2017; Redsell et al., 2011; Turner et al., 2016; Visser et al., 2008) |
| Afraid that discussing the weight of the child will damage the child emotionally | (Edvardsson et al., 2009; Steele et al., 2011; Sheldon et al., 2006) |
| Difficult to make children and parents realize what the impact is of overweight/obesity on health | (Mikhailovich and Morrison, 2007) |
| Negative experiences with discussing weight and lifestyle | (Steele et al., 2011) |
| Expectation that the child and/or parent will react negatively | (Edvardsson et al., 2009; Steele et al., 2011; Sheldon et al., 2006) |
| Discussing weight could stand in the way of having a good relationship with the child or parent | (Edvardsson et al., 2009; McPherson et al., 2017; Redsell et al., 2011; Steele et al., 2011) |
| Discussing weight could be perceived as a negative judgement about the whole family | (McPherson et al., 2017) |
| Parents with overweight or obesity | (Edvardsson et al., 2009; Jackson et al., 2002) |
| Insufficient knowledge about the causes of overweight and obesity | (Holt et al., 2011; Steele et al., 2011; Turner et al., 2016; Visser et al., 2008) |

**Supplementary table 1.** Overview of the literature as basis for the questionnaire

**References**

Brink-Melis WJ, Derksen ER, Westerman MJ, et al. (2012) The local implementation of a chronic disease management model for childhood overweight and obesity. *Obes Facts* 5(5): 766-775.

Edvardsson K, Edvardsson D and Hornsten A (2009) Raising issues about children's overweight--maternal and child health nurses' experiences. *J Adv Nurs* 65(12): 2542-2551.

Freedman MR and Stern JS (2004) The role of optimal healing environments in the management of childhood obesity. *J Altern Complement Med* 10 Suppl 1: S231-244.

Gerards SM, Dagnelie PC, Jansen MW, et al. (2012) Barriers to successful recruitment of parents of overweight children for an obesity prevention intervention: a qualitative study among youth health care professionals. *BMC Fam Pract* 13: 37.

Holt N, Schetzina KE, Dalton WT, et al. (2011) Primary Care Practice Addressing Child Overweight and Obesity: A Survey of Primary Care Physicians at Four Clinics in Southern Appalachia. *Southern Medical Journal* 104(1): 14-19.

Jackson Y, Dietz WH, Sanders C, et al. (2002) Summary of the 2000 Surgeon General's listening session: toward a national action plan on overweight and obesity. *Obes Res* 10(12): 1299-1305.

Jelalian E, Boergers J, Alday CS, et al. (2003) Survey of physician attitudes and practices related to pediatric obesity. *Clin Pediatr (Phila)* 42(3): 235-245.

Kubik MY, Story M and Davey C (2007) Obesity prevention in schools: current role and future practice of school nurses. *Prev Med* 44(6): 504-507.

McPherson AC, Swift JA, Peters M, et al. (2017) Communicating about obesity and weight-related topics with children with a physical disability and their families: spina bifida as an example. *Disabil Rehabil* 39(8): 791-797.

Mikhailovich K and Morrison P (2007) Discussing childhood overweight and obesity with parents: a health communication dilemma. *Journal of Child Health Care* 11(4): 311-322.

Moyers P, Bugle L and Jackson E (2005) Perceptions of school nurses regarding obesity in school-age children. *J Sch Nurs* 21(2): 86-93.

Price JH, Desmond SM and Stelzer CM (1987) Elementary school principals' perceptions of childhood obesity. *J Sch Health* 57(9): 367-370.

Quelly SB (2014) Childhood obesity prevention: a review of school nurse perceptions and practices. *J Spec Pediatr Nurs* 19(3): 198-209.

Redsell SA, Atkinson PJ, Nathan D, et al. (2011) Preventing childhood obesity during infancy in UK primary care: a mixed-methods study of HCPs' knowledge, beliefs and practice. *BMC Fam Pract* 12: 54.

Sheldon LK, Barrett R and Ellington L (2006) Difficult communication in nursing. *J Nurs Scholarsh* 38(2): 141-147.

Steele RG, Wu YP, Jensen CD, et al. (2011) School nurses' perceived barriers to discussing weight with children and their families: a qualitative approach. *J Sch Health* 81(3): 128-137.

Turner GL, Owen S and Watson PM (2016) Addressing childhood obesity at school entry: Qualitative experiences of school health professionals. *J Child Health Care* 20(3): 304-313.

Visser F, Hiddink G, Koelen M, et al. (2008) Longitudinal changes in GPs' task perceptions, self-efficacy, barriers and practices of nutrition education and treatment of overweight. *Fam Pract* 25 Suppl 1: i105-111.
