# Supplementary material for: Talking with pediatric patients with overweight or obesity and their parents: self-rated self-efficacy and perceived barriers of Dutch healthcare professionals from seven disciplines
Source: BMC Health Serv Res. 2022 Oct 6;22:1236. doi: 10.1186/s12913-022-08520-2 (PMC9541008; doi:10.1186/s12913-022-08520-2)
Supplement: Supplementary file 2 — Additional file 2: [file 12913_2022_8520_MOESM2_ESM.docx]

**Supplementary table 2a** Interdisciplinary differences in scores on self-efficacy

| **Self-efficacy** | **Pediatricians** | **Dieticians** | **YHCPs** | **Mental health prof.** | **YHCNs** | **Physiotherapists** | **GPs** |
| --- | --- | --- | --- | --- | --- | --- | --- |
| **Pediatricians** | - | -0.7 | -0.7 | **-0.9*** | **-1.1*** | **-1.2*** | **-1.3*** |
| **Dieticians** | - | - | -0.1 | -0.3 | -0.4 | -0.6 | -0.6 |
| **YHCPs** | - | - | - | -0.2 | -0.4 | -0.5 | -0.6 |
| **Mental health prof.** | - | - | - | - | -0.2 | -0.3 | -0.4 |
| **YHCNs** | - | - | - | - | - | -0.2 | -0.2 |
| **Physiotherapists** | - | - | - | - | - | - | 0.1 |
| **GPs** | - | - | - | - | - | - | - |

Presented numbers are the mean differences from Post-hoc analyses. Significant differences (p<0.05) determined using ANOVA with Bonferroni correction, are marked with an asterisk.

**Supplementary table 2b** Interdisciplinary differences in number of perceived barriers

| **Perceived Barriers** | **Pediatricians** | **Dieticians** | **YHCPs** | **Mental health prof.** | **YHCNs** | **Physiotherapists** | **GPs** |
| --- | --- | --- | --- | --- | --- | --- | --- |
| **Pediatricians** | - | **1.5*** | **2.4*** | 0.6 | **1.9*** | **2.1*** | **2.2*** |
| **Dieticians** | - | - | 0.8 | 0.9 | 0.4 | 0.6 | 0.6 |
| **YHCPs** | - | - | - | **-1.8*** | -0.5 | -0.3 | 0.2 |
| **Mental health prof.** | - | - | - | - | **1.3*** | **1.5*** | 1.5 |
| **YHCNs** | - | - | - | - | - | 0.2 | 0.2 |
| **Physiotherapists** | - | - | - | - | - | - | 0.1 |
| **GPs** | - | - | - | - | - | - | - |

Presented numbers are the mean differences from Post-hoc analyses. Significant differences (p<0.05) determined using ANOVA with Bonferroni correction, are marked with an asterisk.

**Supplementary table 2c** Interdisciplinary differences in avoidance of the topic

| **Perceived Barriers** | **Pediatricians** | **Dieticians** | **YHCPs** | **Mental health prof.** | **YHCNs** | **Physiotherapists** | **GPs** |
| --- | --- | --- | --- | --- | --- | --- | --- |
| **Pediatricians** | - | -7.6% | -7.5% | 4.7% | -10.6% | 17.1% | **47.1%*** |
| **Dieticians** | - | - | 0.1% | 12.3% | -3.0% | **24.7%*** | **54.7%*** |
| **YHCPs** | - | - | - | 12.2% | -3.1% | **24.6%*** | **54.6%*** |
| **Mental health prof.** | - | - | - | - | **-15.3*** | 12.4% | **42.4%*** |
| **YHCNs** | - | - | - | - | - | **27.7%*** | **57.7%*** |
| **Physiotherapists** | - | - | - | - | - | - | 30.0% |
| **GPs** | - | - | - | - | - | - | - |

Presented percentages are the interdisciplinary difference of the proportion that do not discuss weight in ≥50% of cases. Significant differences (p<0.05) from Chi Square analyses are marked with an asterisk.
